# Primary Spinal Epidural/Extramedullary Ewing Sarcoma in Young Female Patients

**DOI:** 10.5435/JAAOSGlobal-D-19-00072

**Published:** 2019-11-27

**Authors:** Amanda N. Fletcher, Joanne Abby M. Marasigan, Stephen V. Hiatt, John T. Anderson, Eugenio M. Taboada, Richard M. Schwend

**Affiliations:** From the Department of Orthopaedic Surgery, Duke University Medical Center, Durham, North Carolina (Dr. Fletcher); Department of Orthopaedic Surgery, University of Missouri Kansas City School of Medicine, Kansas City, Missouri (Dr. Marasigan); the Department of Orthopaedic Surgery, Kansas City Bone and Joint Clinic, Kansas City, Missouri (Dr. Hiatt); the Department of Orthopaedic Surgery, Kansas City, Missouri (Dr. Anderson and Dr. Schwend), Children's Mercy Hospital, Kansas City; and the Department of Pathology and Laboratory Medicine, Children's Mercy Hospital, Kansas City, Missouri (Dr. Taboada).

## Abstract

Primary spinal epidural/extramedullary ewing sarcoma (ES) is a rare extraosseous lesion. Extraosseous ES has a similar demographic as osseous ES, primarily affecting adolescents and young adults and male propensity. Reported 5-year survival is 0% to 37.5% for spinal extraosseous ES.

**Methods:**

Two girls, 19 and 14 months old, presented with progressive lower extremity paraplegia and incontinence. Both had a compressive epidural/extramedullary mass without metastases and underwent decompression with multilevel laminectomy and tumor excision. Primary spinal epidural/extramedullary ES was diagnosed.

**Results:**

Case 1 received 34 weeks of chemotherapy and radiation therapy, and case 2 received 14 cycles of chemotherapy and autologous stem cell rescue without radiation therapy. After more than 5- and 8-year follow-up, case 1 and case 2 are walking and disease-free, respectively.

**Conclusion:**

These cases are the youngest presentation reported for primary spinal epidural/extramedullary ES and suggest that toddlers have a better prognosis for survival than older children and adolescents.

Ewing sarcoma (ES) is a malignancy of the bone and rarely soft tissues. Although osseous ES is the second most common primary bone tumor in pediatric patients,^1^ extraosseous ES is rare, usually arising from the chest wall, paravertebral muscles, extremities, pelvis, and retroperitoneal space.^2,3^ Primary spinal epidural/extramedullary ES is a very rare lesion. Extraosseous ES has a similar demographic as osseous ES, primarily affecting adolescents and young adults with a male sex propensity. We report two cases of primary spinal epidural/extramedullary ES in young female patients who presented with neurologic compromise.

## Case 1

A 19-month-old white female patient presented with a 2-week history of refusal to bear weight, lower extremity pain, and acute onset of bowel and bladder incontinence. She was previously walking for 6 months. Her medical history was normal. Examination revealed no response to stimuli of her feet, decreased tone in her bilateral lower extremities and 3/5 strength with hip flexion and knee extension. She could not plantar or dorsiflex her ankles or toes. She exhibited 2/4 knee reflexes, absent ankle reflexes, negative Babinski signs, and no clonus. Rectal tone was absent with visible bladder incontinence.

**Figure 1:**
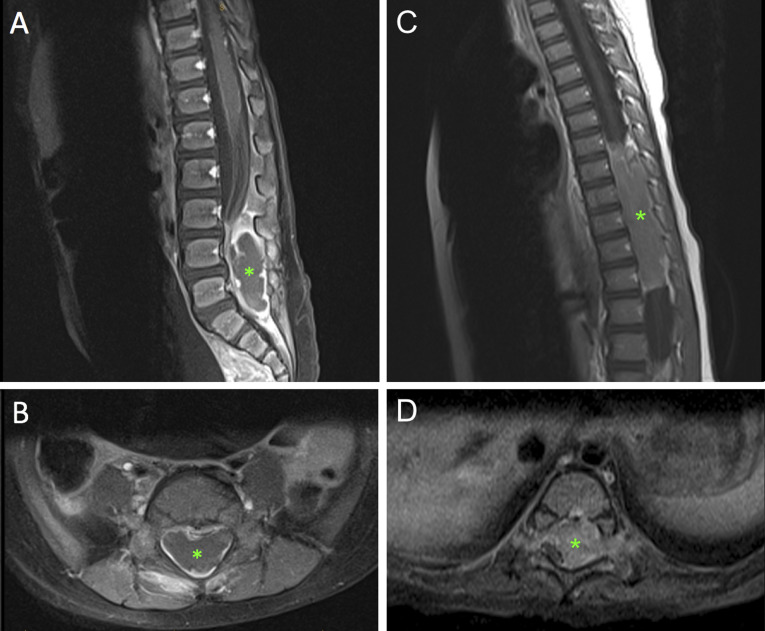
(**A** and **B**) Case 1 T1-weighted postcontrast preoperative sagittal and axial spinal MRI. Asterisk indicates a 5.3 x 1.5 x 2.3-cm epidural mass extending from L3 to S1 with foraminal involvement. The mass has a rim-enhancing component causing notable ventral displacement of the cauda equine. (**C** and **D**) Case 2 T1-weighted postcontrast preoperative sagittal and axial spinal MRI. Asterisk indicates a mass extending from T7 to 12 with dorsal and right-sided displacement of the spinal cord with bilateral foraminal extension.

**Figure 2:**
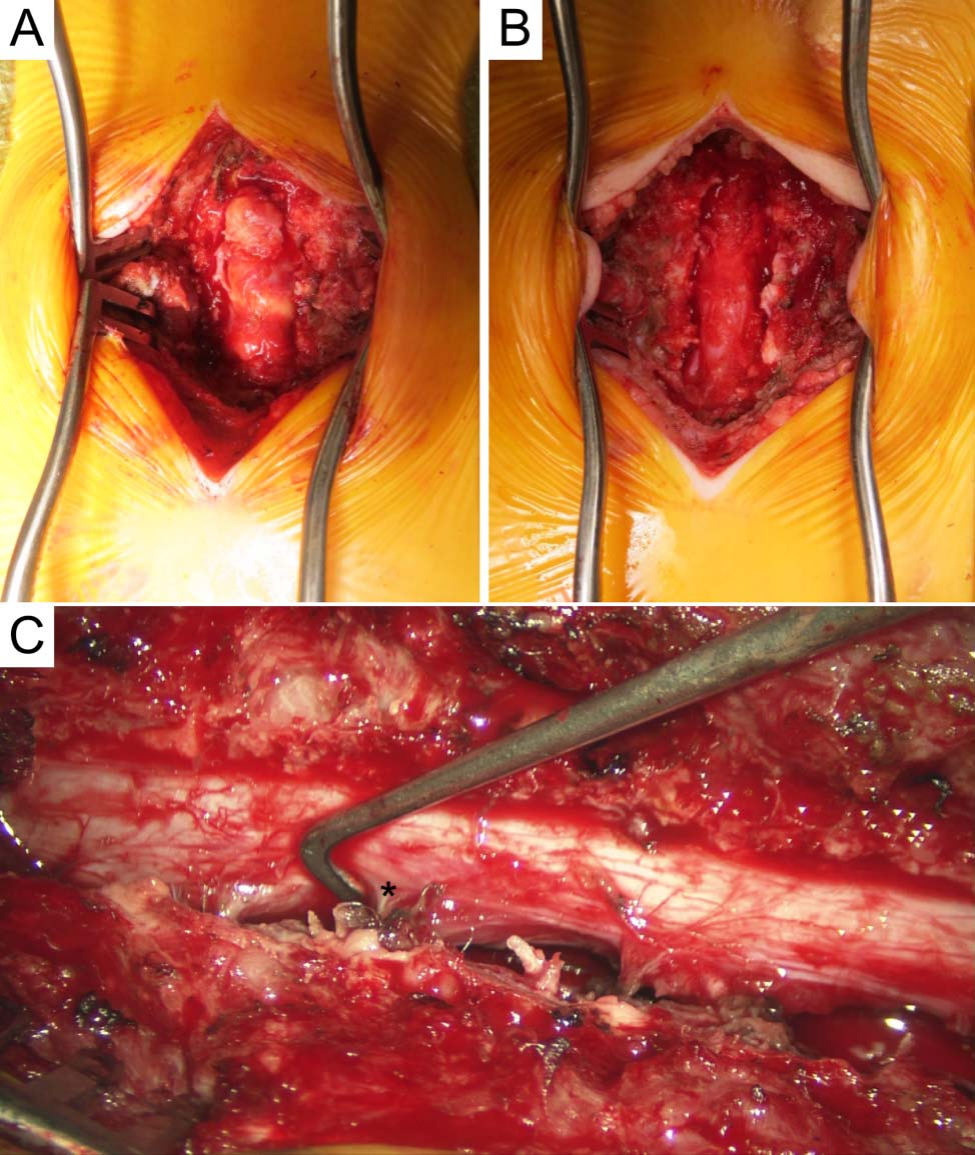
(**A**) Case 1 intraoperative image of mass pre-excision after L3-S1 laminectomies. Mass was soft with distinct borders, light yellow in coloration, and homogenous. (**B**) Mass was completely excised off the dura, and the foraminal portions of the mass were removed without difficulty. (**C**) Case 2 intraoperative image after resection. T7-L1 laminectomies were done revealing a vascular, soft, extradural tumor. Asterisk marks the left lower thoracic nerve after resection of mass.

A total spine MRI with and without contrast revealed an epidural/extramedullary well-circumscribed mass extending from L3 to S1 with spinal cord effacement (Figure 1, A and B). Mass was isointense on T1, hyperintense on T2, and peripherally enhanced with contrast. No additional imaging was obtained, and the patient underwent urgent surgical decompression. Laminectomies were done from L3 to S1, revealing an extradural/extramedullary mass which was marginally excised without disruption of the dura (Figure 2, A and B).

**Figure 3:**
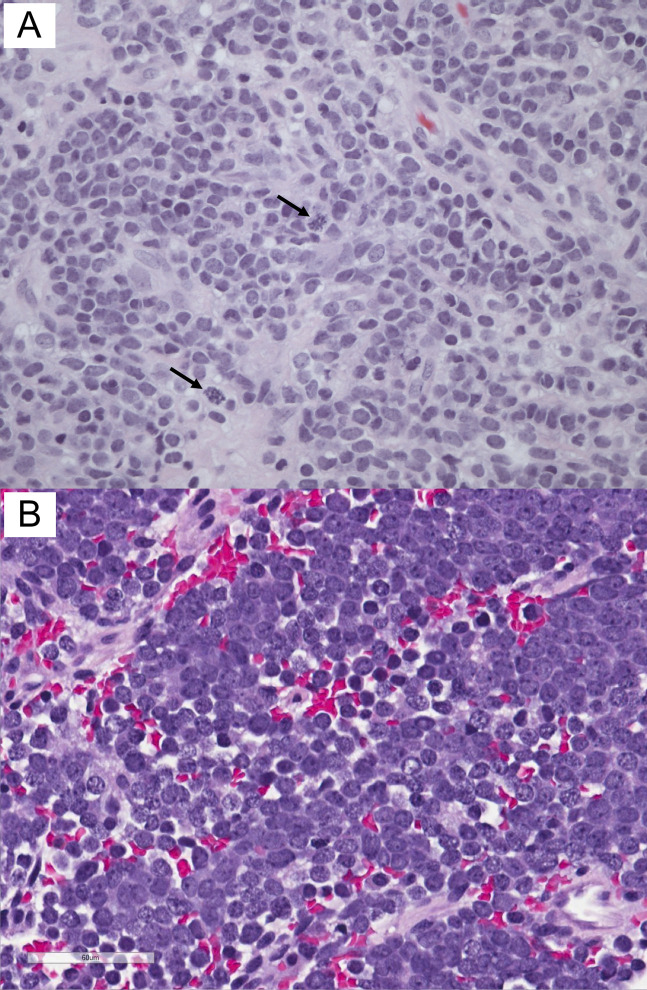
(**A**) Case 1 histology shows undifferentiated small blue cells consisting of round to oval hyperchromatic nuclei, no nucleoli, and scant cytoplasm. Cells were arranged in sheets with strands of collagenous stroma, forming vaguely lobular architecture. Arrows indicate mitoses. (**B**) Case 2 histology shows sheets and small clusters of small round blue cells with background hemorrhage and necrosis. The tumor diffusely infiltrates the surrounding fibrous tissue (hematoxylin and eosin stain, ×400).

Histopathologic examination revealed an undifferentiated small blue cell neoplasm (Figure 3, A). Immunohistochemically, the tumor cells were positive for CD99 and vimentin. Fluorescence in situ hybridization analysis showed EWSR1 gene rearrangement (22q12), confirming the diagnosis of ES.

Additional evaluation with bone marrow aspiration, positron emission tomography (PET) scan, and bone scan did not reveal evidence of metastatic disease. She underwent 34 weeks of chemotherapy per the AEWS1031 protocol,^4^ regimen B, and local radiation therapy with a total dose of 5580 cGy. She is currently walking and in remission at her most recent 5-year follow-up. Remaining deficits include neurogenic bowel and bladder, 4/5 strength with bilateral ankle dorsiflexion, diminished sensation in the S1 distribution, and absent knee and ankle reflexes. Pediatric Outcome Data Collecting Instrument Global Functioning Scale standardized and normalized scores were 84 and 36, respectively.

## Case 2

A 14-month-old white female patient presented with a 1-month history of progressive lower extremity weakness and urinary incontinence. She was previously walking for 3 months and regressed to inability to bear weight or stay seated due to trunk weakness. Her medical history was normal. Examination revealed weak withdrawal of her legs to noxious stimuli, minimal spontaneous leg movement, and flaccid tone. She exhibited 0/4 knee reflexes, 1/4 ankle reflexes, positive Babinski sign bilaterally, and bilateral ankle clonus. She had decreased trunk strength and sensibility below T10. Normal rectal tone was present.

A total spine MRI with and without contrast revealed an epidural/extramedullary well-circumscribed mass extending from T7 to T12 with spinal cord displacement. Mass was isointense on T1, hyperintense on T2, and enhanced heterogeneously with contrast (Figure 1, C and D). No additional imaging was obtained. Decompression with T7-L1 laminectomies was done, revealing an extradural/extramedullary mass which was marginally excised without disruption of the dura (Figure 2, C).

Histopathologic examination revealed a small blue round cell tumor (Figure 3, B). Immunohistochemically, the tumor cells were positive for CD99, neuron-specific enolase, and synaptophysin. Fluorescence in situ hybridization analysis showed EWSR1 gene rearrangement (22q12), confirming the diagnosis of ES.

Additional evaluation with CT of the chest-abdomen-pelvis, bone marrow aspiration, PET scan, and bone scan did not reveal evidence of metastatic disease. She underwent 14 cycles of chemotherapy per the AEWS0031 protocol,^5^ regimen B, and autologous stem cell rescue. She is currently walking, active in dance, and in remission at her most recent 8-year follow-up. Remaining deficits include neurogenic bowel and bladder, 4/5 strength in most of her bilateral lower extremity muscle groups, 1/4 knee reflexes, absent ankle reflexes, and positive left Babinski sign. Pediatric Outcome Data Collecting Instrument Global Functioning Scale standardized and normalized scores were 83 and 35, respectively.

## Conclusions

To the best of our knowledge, our two patients at 19 and 14 months old represent the youngest age of presentation reported for primary spinal epidural/extramedullary ES. The peak incidence of extraosseous ES is approximately 20 years old,^2^ with a meta-analysis summarizing less than one-sixth of primary spinal extradural ES occurring in children younger than 10 years.^4,6^

In extraosseous ES, MRI findings show a mass hypointense on T1, hyperintense on T2, and with varying postcontrast enhancement. Histopathology demonstrates numerous small round blue cells with a high nuclear cytoplasmic ratio. Immunohistochemistry is positive for CD99 and may be positive for vimentin, bcl-2, neuron-specific enolase, Leu-7, S-100, synaptophysin, and chromogranin. Immunohistochemistry is also negative for leukocyte common antigen, epithelial membrane antigen, cytokeratin, desmin, smooth muscle actin, and myogenin. Cytogenetic studies with the most common EWSR1 gene rearrangement (22q12) confirm the diagnosis of ES.^3,4^ Although no clear therapeutic protocol exists for patients, these tumors are typically treated with chemotherapy followed by surgery with or without radiation.

Prompt decompression was necessary for these two patients because of progressive neurologic compromise. These favorable outcomes are significant with respect to this tumor's characterization of aggressive growth, rapid progression, local recurrence with invasion of adjacent structures, and dismal prognosis. Reported 5-year survival is only 0% to 37.5% for extraosseous ES around the spinal canal.^3^ Case 1 and case 2 are walking and disease-free after 5- and 8-year follow-up, respectively. Although no conclusions can be made from two patients, it is suggestive that toddlers presenting with primary spinal extradural ES have a better prognosis for survival than older children and young adults.
